# Ampithoidae (Crustacea, Amphipoda) from New Zealand

**DOI:** 10.3897/zookeys.733.14052

**Published:** 2018-01-26

**Authors:** Rachael A. Peart, Anne-Nina Lörz

**Affiliations:** 1 P.O. Box 14901, Kilbirnie, Wellington, 6241, New Zealand; 2 CeNak, Zoologisches Institut und Museum, Martin-Luther-King Platz 3, 20146 Hamburg, Germany

**Keywords:** Algae dweller, new species, Peracarida, New Zealand

## Abstract

Ampithoidae is a family of marine Amphipoda with approximately 230 species, belonging to 16 genera. The family has a worldwide distribution as algal dwellers. So far only five species are known from New Zealand. Recent collections and examination of historic collection material added two new species, which are described herein. An overview of and a key to the New Zealand Ampithoidae is provided.

## Introduction

The family, Ampithoidae, is a broadly distributed group of primarily algal-dwelling amphipods. They have been well described and reported from North and South American-, European-, African-, Australian- and Asian waters. The Ampithoidae record is quite sparse in boreal waters, but not unknown (De Broyer et al. 2007). Globally there are 231 species (Horton et al. 2017). However, despite this diversity, there have only been five species recorded from New Zealand.

The major source of information on New Zealand ampithoid amphipods is J.L. Barnard’s detailed monograph of algal dwelling gammarid amphipods (1972). This paper documents four species of this family all in the genus, *Ampithoe* Leach, 1814. One of these species was originally described by Hurley (1954) (as *Pleonexes lessoniae* Hurley, 1954) and was subsequently designated the type species for the genus *Pseudopleonexes* (Conlan, 1982), two others were new descriptions (*Ampithoe hinatore* and *Ampithoe aorangi*) and the last an undesignated species (*Ampithoe* sp.). Recent work (Hughes and Peart 2015, Peart 2017, and this current work) has expanded and revised this previous research.

Using freshly collected material and type material from the NIWA Invertebrate collection, this paper provides a checklist to the Ampithoids from New Zealand waters, providing a diagnosis for each species and a key to the species of the family. Adding to the count of species from these is the description of two new species, *Exampithoe plumosa* sp. n. and *Pseudopleonexes evensis* sp. n.

## Materials and methods

Recent collections were sampled via snorkelling from *Macrocystis* sp. in Wellington and collected from a large, drifting detached plant of *Durvillaea antarctica* from Otago Harbour. They were immediately preserved in 95% ethanol. Specimens were examined and dissected using a Leica MZ12.5, in Wellington and drawn using a camera lucida attachment. Small appendages (mouthparts, uropods, telson) were temporarily mounted in glycerin and examined and drawn using a compound microscope (Zeiss, in Wellington) fitted with a camera lucida. The body lengths of specimens examined were measured by tracing individual’s mid-trunk lengths (tip of the rostrum to end of telson) using a camera lucida. For scanning electron microscope (SEM) imaging the specimens and appendages were dehydrated through a graduated ethanol series, acetone dried, mounted on studs, coated with gold-palladium and investigated via a SEM LEO1525.

Type material and other material examined is held at the "National Institute of Water and Atmospheric Research Invertebrate Collection at Wellington, New Zealand (NIWA) and the CeNak, Zoological Museum Hamburg.

## Systematics

### Order AMPHIPODA Latreille, 1816

#### Suborder SENTICAUDATA Lowry & Myers, 2013

##### Ampithoidae Boeck, 1870

In New Zealand waters, the fauna of the family Ampithoidae is represented by four genera comprised of seven species, two of which are newly described here.

###### *Ampithoe* Leach, 1814

####### Ampithoe hinatore

Taxon classificationAnimaliaAmphipodaAmpithoidae

J.L. Barnard, 1972

Ampithoe hinatore J.L. Barnard, 1972: 39–42, figs 11–12.

######## Type material.

Holotype: male, 9.0 mm, NIWA 831, station E970, Kaikoura, New Zealand, 42.417°S 173.700°E, intertidal wash of algae, 22 January, 1968.

Paratype: Female, 7.3 mm, 2 specimens, NIWA 832, station E970, Kaikoura, New Zealand, 42.417°S 173.700°E, intertidal wash of algae, 22 January, 1968.

######## Diagnosis.

Male: Eye prominent. Epistome and upper lip, in situ, directed straight down, perpendicular to the head. Lower lip outer plate notched, outer lobe extending past inner lobe. Mandible molar well developed and triturating, palp robust and three-articulate, article three distally rounded. Maxilla 1 palp well developed. Pereopods weakly setose. Gnathopod 1 weakly sexually dimorphic; coxa produced anteriorly; basis anteroventral lobe prominent; propodus subovoid, anterodistal setose lobe absent, palm acute and concave defined by a subacute posterodistal tooth and robust seta; dactylus subequal in length to palm. Gnathopod 2 more robust and slightly longer than gnathopod 1; basis anteroventral lobe large and setose; carpus subtriangular; propodus longer than carpus, propodus broad, anterodistal lobe absent, palm acute, defined by a subacute posterodistal tooth and robust seta; dactylus subequal in length to palm. Pereopods 3 and 4 similar in size and shape; basis slightly expanded and glandular; merus narrow, lobe absent. Pereopod 5 basis rounded; distal articles slender; propodus weakly prehensile. Pereopods 6 and 7 similar, increasing in length; distal articles slender; propodus weakly prehensile. Epimeron 3 posteroventral corner rounded without tooth. Uropod 1, in situ, reaching to the end of uropod 2, peduncle distoventral spur absent. Uropod 2 peduncle rounded lateral distoventral process absent. Uropod 3 broad, peduncle with six distal robust setae; rami short; outer ramus with two recurved robust setae, denticle patch; inner ramus with robust and slender distal setae. Telson subtriangular with reduced, small cusps, denticles absent, with lateral and apical setae.

Female. Similar to male, except: Gnathopods 1–2 palms less excavate.

######## Remarks.

Known only from the type locality, Kaikoura on the New Zealand, South Island north-eastern coast. J.L. Barnard (1972) recorded only three specimens, whilst Fenwick (1976) also noted the presence of this species in a wave exposure study, but in relatively small abundances (16 out of around 60,000 individuals). Barnard (1972) notes that it has a similar morphology to *Ampithoe waialua* Barnard, 1970 from Hawaii, which is also similar to the *Ampithoe ramondi* Audouin, 1826 complex of species. Lowry (1974) refers to the presence of *Ampithoe hinatore* from Kaikoura, however, these are not new records just repeats from Barnard’s paper.

######## Distribution.

Kaikoura, South Island, New Zealand

###### *Exampithoe* K.H. Barnard, 1925

####### Exampithoe plumosa sp. n.

Taxon classificationAnimaliaAmphipodaAmpithoidae

http://zoobank.org/9ABFEAC2-509F-42C0-936A-C3FAABB215B2

Figs 1 , 2 , 3 , 4

######## Type material.

Holotype, male, 10 mm, NIWA 121270, KH-NZ1-9, from drifting *Durvillaea antarctica* raft from near Taiaroa Head, inside Otago Harbour, Dunedin, New Zealand, 45°46'19"S, 170°43'30"E, 0 m depth, 22 January 2010, J. Waters.

Paratypes: Female, 7 mm, NIWA 121269, Male, 9 mm, ZMH K-46915, KH-NZ1-9, from drifting *Durvillaea antarctica* raft near Taiaroa Head, inside Otago Harbour, Dunedin, New Zealand, 45°46'19"S, 170°43'30"E, 0 m depth, 22 January 2010, J. Waters.

######## Diagnosis.

Male: Eye prominent. Antennae similar length to each other. Antenna 2 peduncular articles robust but not elongated. Epistome and upper lip, in situ, directed straight down, perpendicular to the head. Lower lip outer plate entire. Mandible molar well developed and triturating; palp slender and three-articulate, article three distally rounded. Maxilla 1 palp moderately developed. Pereopods setose with plumose setae. Gnathopod 1 robust and sexually dimorphic, coxa slightly produced anteriorly, basis anteroventral lobe prominent and setose, propodus subrectangular, anterodistal setose lobe absent, palm acute and concave, defined by a very small rounded posterodistal tooth and a large robust seta; dactylus shorter than palm. Gnathopod 2 slender and slightly longer than gnathopod 1, sexually dimorphic; basis anteroventral lobe medium sized and setose; carpus subovoid; propodus subequal in length to carpus, propodus narrow, anterodistal lobe absent, palm acute, midpalmar tooth/corner present, defining posterodistal tooth absent, robust seta present; dactylus shorter than palm length. Pereopods 3 and 4 similar in size and shape, basis expanded and glandular; merus slightly expanded, lobe present. Pereopod 5 basis ovoid; distal articles slender; propodus weakly prehensile. Pereopods 6–7 similar lengths; merus and carpus broader than propodus; propodus weakly prehensile.

Epimeron 3 posteroventral corner rounded without tooth. Uropod 1, in situ, reaching to the end of uropod 2, peduncle distoventral spur absent. Uropod 2 peduncle rounded lateral distoventral process absent. Uropod 3 broad, peduncle with two distal robust setae; rami very short, outer ramus with two recurved robust setae, patch of denticles; inner ramus with just slender distal setae. Telson subrectangular, cusps absent, light denticles present, with lateral and apical setae.

Female. Similar to male except for gnathopod 1 merus lobe reduced and weakly setose, carpus more slender than male and less setose and subequal in length to the propodus; propodus narrow, weakly setose, palm convex, not sculptured.

**Figure 1.**
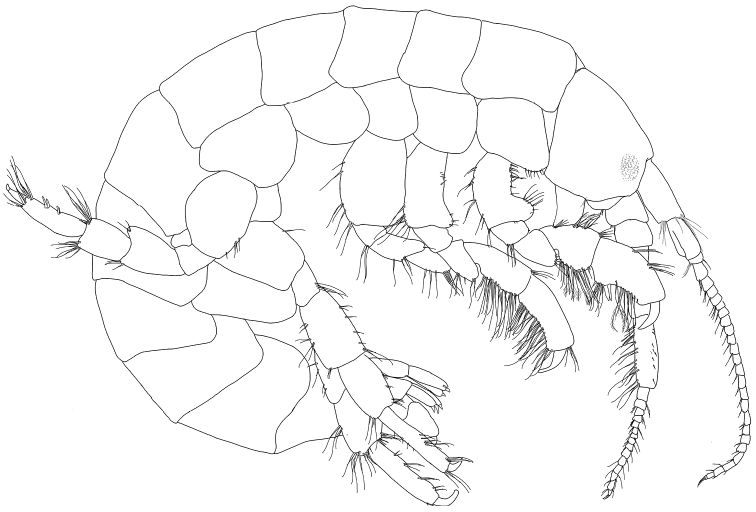
*Exampithoe plumosa* sp. n., holotype, male, 10 mm, NIWA 121270, Otago Harbour, New Zealand.

**Figure 2.**
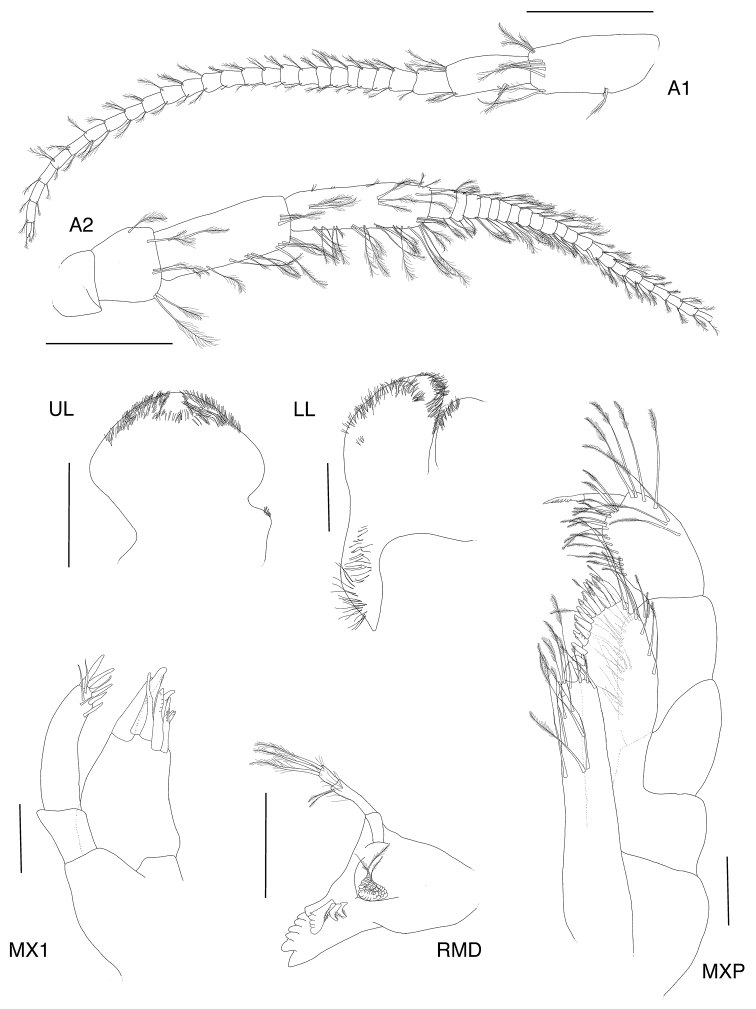
*Exampithoe plumosa* sp. n. holotype, male, 10 mm, NIWA 121270, Otago Harbour, New Zealand. Scale bars: 0.5 mm (**A1–2**); 0.1 mm (**MX1, MXP, LL**); 0.2 mm (**UL, MD**).

**Figure 3.**
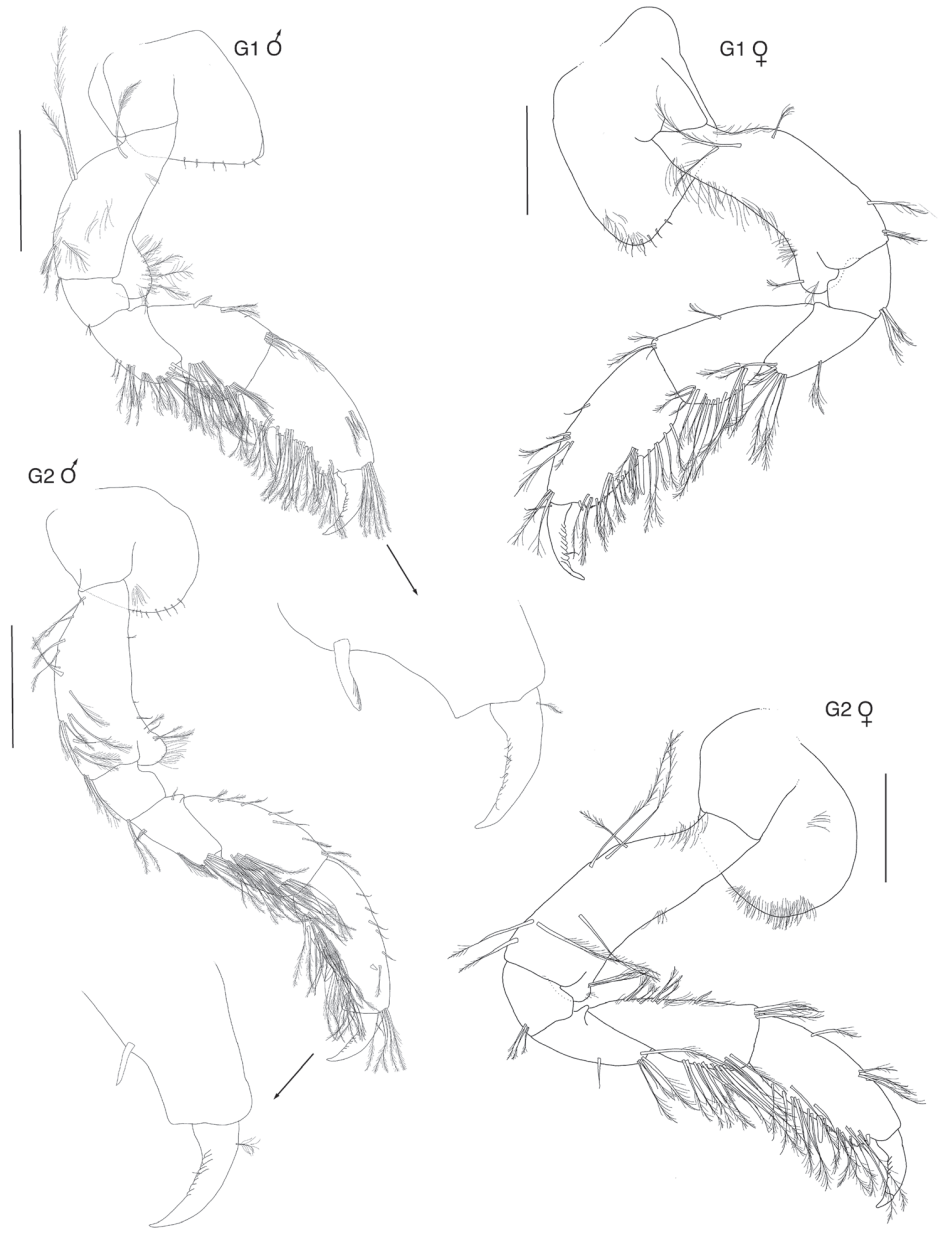
*Exampithoe plumosa* sp. n. holotype, male, 10 mm, NIWA 121270, paratype, female, 7 mm, NIWA 121269, Otago Harbour, New Zealand. Scale bars: 0.5 mm (male **G1–2**); 0.2 mm (female **G1–2**).

**Figure 4.**
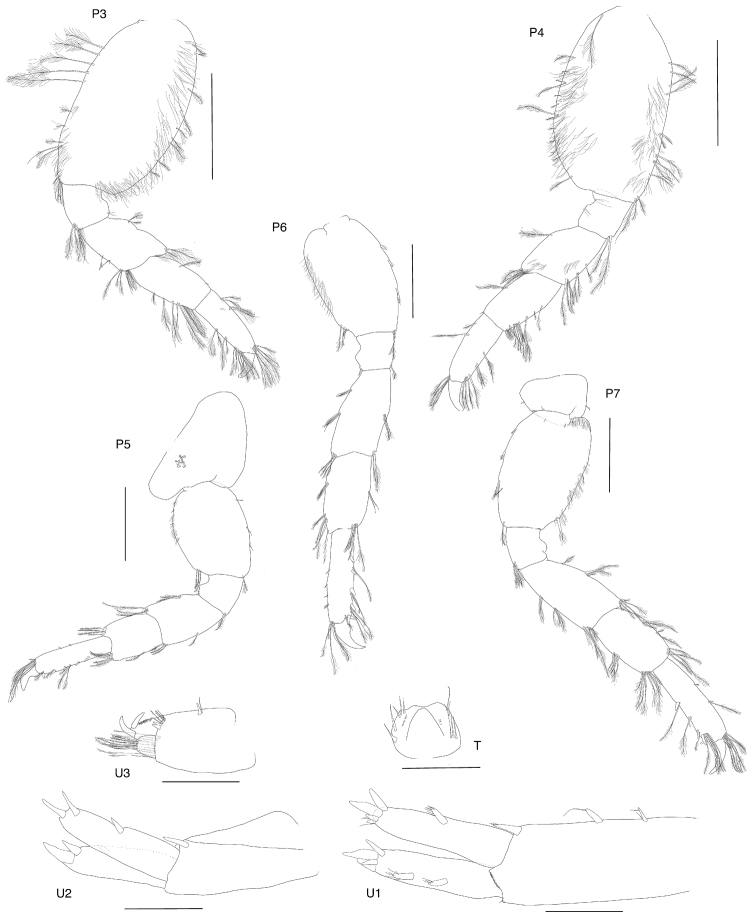
*Exampithoe plumosa* sp. n. holotype, male, 10 mm, NIWA 121270, Otago Harbour, New Zealand. Scale bars: 0.5 mm (**P3–7**); 0.2 mm (**U1–3, T**).

######## Etymology.

Named plumosa, a derivative of the latin plumosus meaning feathered, referring to the feathered nature of majority of the setae present on the type material.

######## Remarks.

This is an interesting species for a couple of reasons. Firstly, it is the first record of this genus from the South Island of New Zealand. The only other *Exampithoe* species recorded from New Zealand is *Exampithoe taylori* Hughes & Peart, 2015. These two species are recorded from almost opposite ends of the country with over 1000 km between them and situated on water bodies influenced by different currents and geophysical history. Though the two species have some similarities, such as the shape of gnathopod 2 propodus (narrow, palm with a subquadrate midmedial tooth), the shape of the lower lip outer plate (entire), similar setation and shape of uropod 3 (broad peduncle, small rami, 2 distal peduncular robust setae, 1 marginal robust seta), there are also a number strong differences that give the necessity of these being separate species. These differences include: the shape and length of the antennae (A1 and A2 similar length in *E. plumosa* sp. n. A1 shorter than A2 in *E. taylori*. Antenna 2 peduncular articles are robust but not elongated in *E. plumosa* sp. n. and are robust but considerably elongated in *E. taylori*); the shape and size of gnathopod 1 (robust with shortened articles, propodus ovoid to subrectangular, palm excavate with small posterodistal tooth, subquadrate predactylus tooth and large defining robust seta in *E. plumosa* n.sp. and narrow with elongated articles, propodus subrectangular, palm convex, no defining tooth or predactylus tooth or robust seta in *E. taylori*). The other main difference between the two species is the majority of the setae on every appendage of *E. plumosa* are feathered (plumose) giving this animal a strongly fuzzy look. Whilst *E. taylori* has numerous setae on the appendages, very few are plumose.

The second interesting aspect of the discovery of this species is that the specimens were collected from a kelp raft. While many organisms can be found on floating or rafting macroalgae, ampithoids are only occasionally recorded rafting (Thiel and Gutow 2005) but are not obligate rafters. As there are no other records of this species, it cannot be inferred whether it is an obligate rafter or not. The kelp was determined to have been drifting in the water for around five weeks (determined by the stage of goose barnacle settlement - Waters et al. in press) and probably originating from southern New Zealand. This is the first record of an ampithoid in this area of New Zealand.

######## Distribution.

Only known from the type locality, Otago Harbour, South Island, New Zealand.

####### Exampithoe taylori

Taxon classificationAnimaliaAmphipodaAmpithoidae

Hughes & Peart, 2015

Exampithoe taylori Hughes & Peart, 2015: 563–566, figs 4–6.
Ampithoe sp. – J.L Barnard, 1972: 45, fig. 15.

######## Type material.

Holotype: male, 11.5 mm, 4 slides, NIWA 94663, Leigh, New Zealand, 36°17'28.76"S, 174°48'10.81"E, coll. R. Taylor, 16 September 2002. Paratypes: 2 males, AM P. 88414, same location as holotype.

######## Additional material examined.

NIWA 94664, 1 male, 9 mm; NIWA 94665, 1 female, 8 mm; NIWA 94666, 1 female, 7 mm; NIWA 94667, 1 female, 10 mm; NIWA 94668, 1 female, 8 mm; NIWA 94669, 1 male, 8 mm; NIWA 94670, 1 male, 7 mm; AM P.92556, 11 females, 15 males, Nordic Cove, Leigh, New Zealand, 36°17'28.76"S, 174°48'10.81"E, 1–3 m, on *Dictyota kunthii*, coll. Richard Taylor, 31 Dec 2009. NIWA 7022, 1 female, 6.4 mm, station E979, Huaroa Pt. Whangaparaoa Peninsula, Auckland Province, New Zealand, 36.59°S 175.84°E, shore collection, coll. J.L. Barnard, 16 Feb 1968.

######## Diagnosis.

Male: Eye prominent. Antenna 1 slightly shorter than antenna 2. Epistome and upper lip, in situ, directed straight down, perpendicular to the head. Lower lip outer plate entire. Mandible molar reasonably well developed and triturating, palp slender and three-articulate, article three distally rounded. Maxilla 1 palp well developed. Pereopods weakly setose. Gnathopod 1 weakly sexually dimorphic; coxa slightly produced anteriorly; basis anteroventral lobe prominent; propodus subrectangular, anterodistal setose lobe absent, palm acute and convex, defining tooth and robust seta absent; dactylus subequal in length to palm. Gnathopod 2 slightly narrower and slightly longer than gnathopod 1, weakly sexually dimorphic; basis anteroventral lobe medium sized and setose; carpus elongated, subtriangular; propodus shorter than carpus, propodus narrow, anterodistal lobe absent, palm acute and convex, without defining tooth, but with a defining robust seta; dactylus subequal in length to palm. Pereopods 3 and 4 similar in size and shape; basis expanded and glandular; merus slightly expanded, lobe small and reduced. Pereopod 5 basis ovoid; distal articles slender; propodus weakly prehensile. Pereopods 6 and 7 similar in shape to each other, increasing in length; distal articles slender; propodus weakly prehensile. Epimeron 3 posteroventral corner rounded without tooth. Uropod 1, in situ, reaching to the end of uropod 2, peduncle distoventral spur absent. Uropod 2 peduncle without rounded lateral distoventral process. Uropod 3 broad, peduncle with two distal robust setae and 1 marginal robust seta; rami short; outer ramus with two recurved robust setae, lateral patch of denticles; inner ramus with robust and slender distal setae. Telson subtriangular with reduced, small cusps, denticles absent, with lateral and apical setae.

Female: Similar to male.

######## Remarks.

Known from northern New Zealand only. This species has similarities, but more significant differences to *E. plumosa* sp. n. These differences are discussed in the remarks above. Material examined from J.L. Barnard (1972) identified as *Ampithoe* sp. has been examined and identified as *Exampithoe taylori*.

######## Distribution.

Leigh and Whangaparaoa, North Island, New Zealand.

###### *Pseudopleonexes* Conlan, 1982

####### Pseudopleonexes evensis sp. n.

Taxon classificationAnimaliaAmphipodaAmpithoidae

http://zoobank.org/29497044-6CC3-4D94-BC16-6BB85E6C0404

Figs 5 , 6 , 7

Ampithoe (Pleonexes) lessoniae .—Barnard 1972: 44, figs 13–14.

######## Type material.

Holotype, 9 mm, male, NIWA 121291, from algal washings, 0. 5 m, Eve Bay, Wellington, New Zealand, 41°19'58"S, 174°49'39"E, coll. R. Peart and J. Peart, 29 Nov 2016.

Paratype, female, 7 mm, NIWA 121292. Paratypes, female and male, ZMH K-46614, Same collection data as the holotype.

######## Other material examined.

6 specimens, male, female and juveniles, NIWA 121894, same collection data as the holotype. Male, 1 specimen, NIWA 7024, E797, from intertidal algal washings, 0.5 m, Huaroa Pt, Whangaparaoa, New Zealand, 36°35.7'S 175°50.14'E, coll J. L. Barnard, 16 Feb 1968.

######## Diagnosis.

Male: eye prominent. Antennae damaged in type material (Barnard, 1972 material antenna 1 longer than antenna 2). Epistome and upper lip, in situ, directed posteriorly at an angle of around 45°. Lower lip outer plate weakly notched, lobes of even size. Mandibular molar reduced and triturating, palp reduced, 2 articles, article 2 distally rounded. Maxilla 1 palp 2-articulate, reduced and slender. Gnathopods densely setose, pereopods weakly setose. Gnathopod 1 slender, sexually dimorphic; coxa slightly produced anteroventrally, basis anterodistal lobe medium and slightly upturned, bearing three slender setae; propodus subrectangular and narrow, with a strongly setose anterodistal lobe, palm acute, short, entire, without posterodistal tooth defining palm, without defining robust seta; dactylus greatly overreaching palm. Gnathopod 2 robust and longer than gnathopod 1, with long, dense simple setae on margins; basis anterodistal lobe medium and rounded, with four slender to robust setae on lobe margin, five robust setae on anterior margin of basis; carpus subtriangular; propodus longer than carpus; propodus broad, ovoid, produced into an anterodistally setose lobe; palm acute, excavate, with small subacute posterodistal tooth defining palm, with one defining robust seta; dactylus subequal in length to palm. Pereopods 3–4 similar in size and shape; basis expanded and glandular; merus expanded to form an acute lobe. Pereopod 5 basis circular, distal articles broad, propodus prehensile. Pereopod 6 shorter than pereopod 7, merus and carpus similar width to propodus, propodus prehensile. Epimeron 3 posteroventral corner rounded, no tooth. Uropod 1, in situ, reaching to end of uropod 2 peduncle; peduncle distoventral spur absent. Uropod 2 peduncle with rounded lateral distoventral process. Uropod 3 broad, peduncle with distal robust setae absent, rami short; outer ramus with two recurved robust setae; patch of denticles; inner ramus with just slender distal setae. Telson subtriangular, with two large recurved cusps and with 4 slender setae per lobe.

Female. Similar to the male except for gnathopod 1 basis anteroventral lobe reduced, merus is weakly setose; carpus shorter than propodus, carpal lobe slightly truncated; propodus weakly setose, anterodistal lobe reduced. Gnathopod 2 weakly setose, carpus shorter than propodus, carpal lobe rounded; propodus anterodistal lobe reduced, palm weakly excavate, posterodistal tooth reduced. Uropod 2 peduncle laterodistal projection absent.

**Figure 5.**
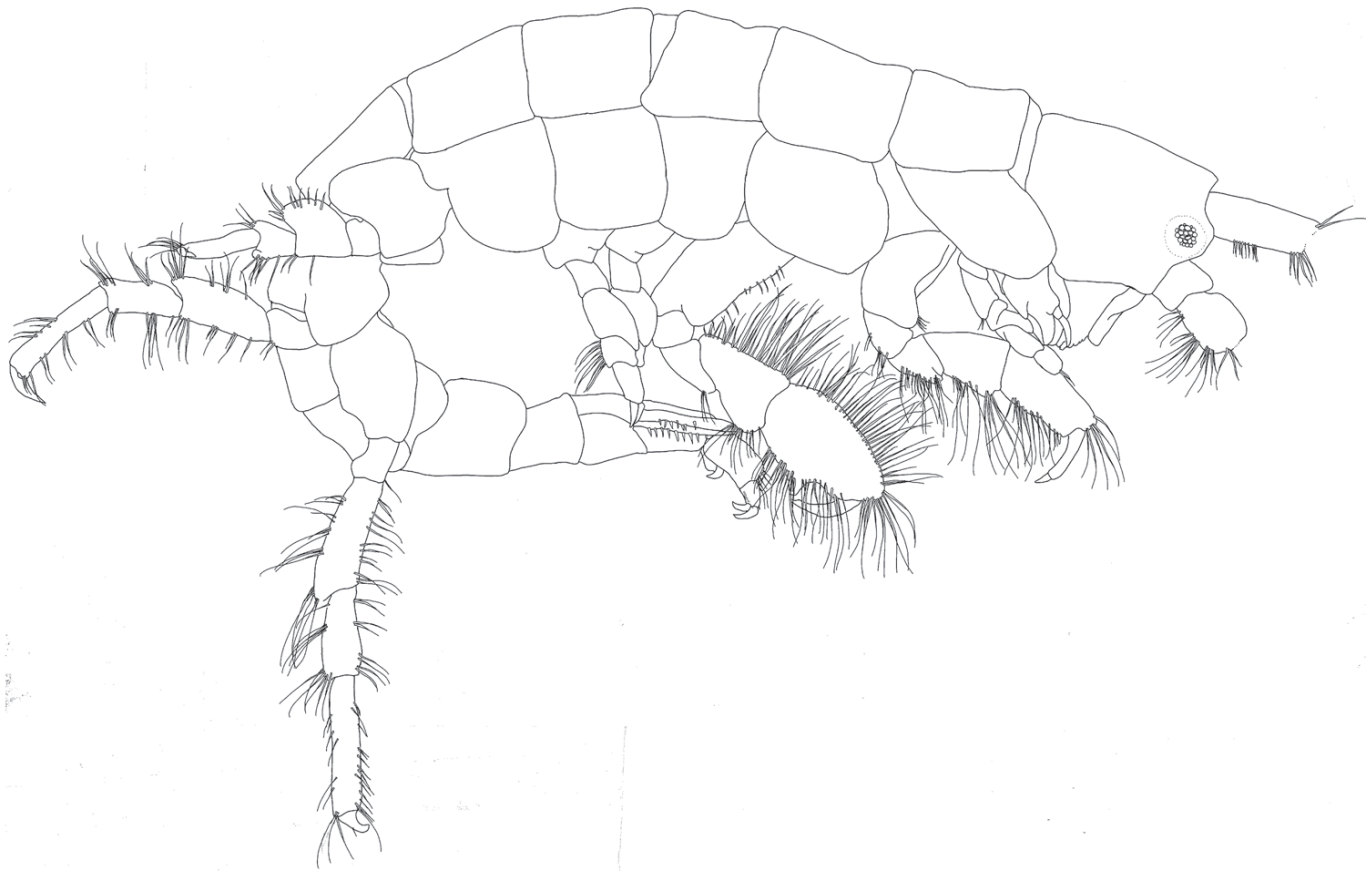
*Pseudopleonexes evensis* sp. n. holotype, 9 mm, male, NIWA 121291, Eve Bay, Wellington, New Zealand.

**Figure 6.**
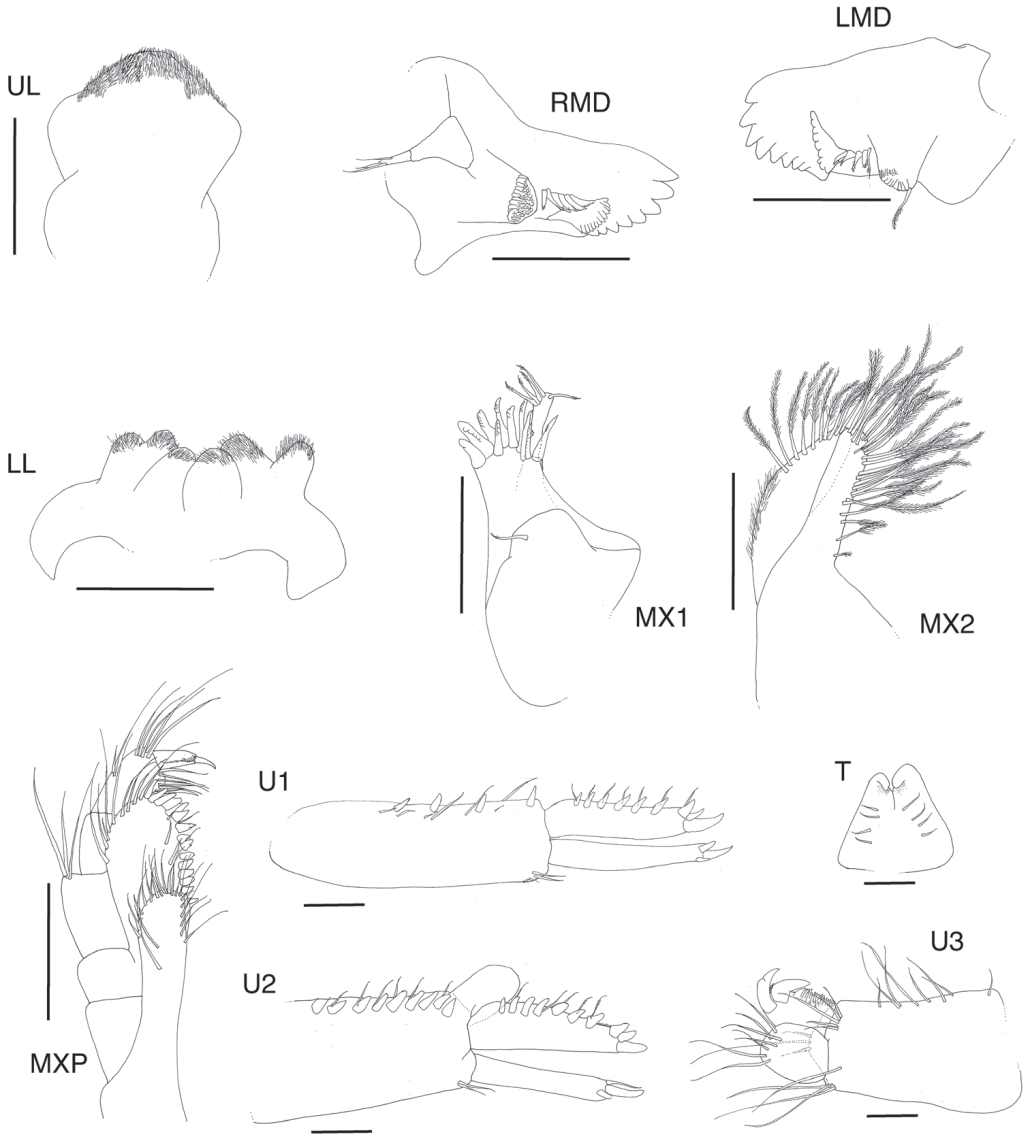
*Pseudopleonexes evensis* sp. n. Holotype, 9 mm, male, NIWA 121291, Eve Bay, Wellington, New Zealand. Scale bars: 0.5 mm (mouthparts), 0.1 mm (**U1–3, T**).

**Figure 7.**
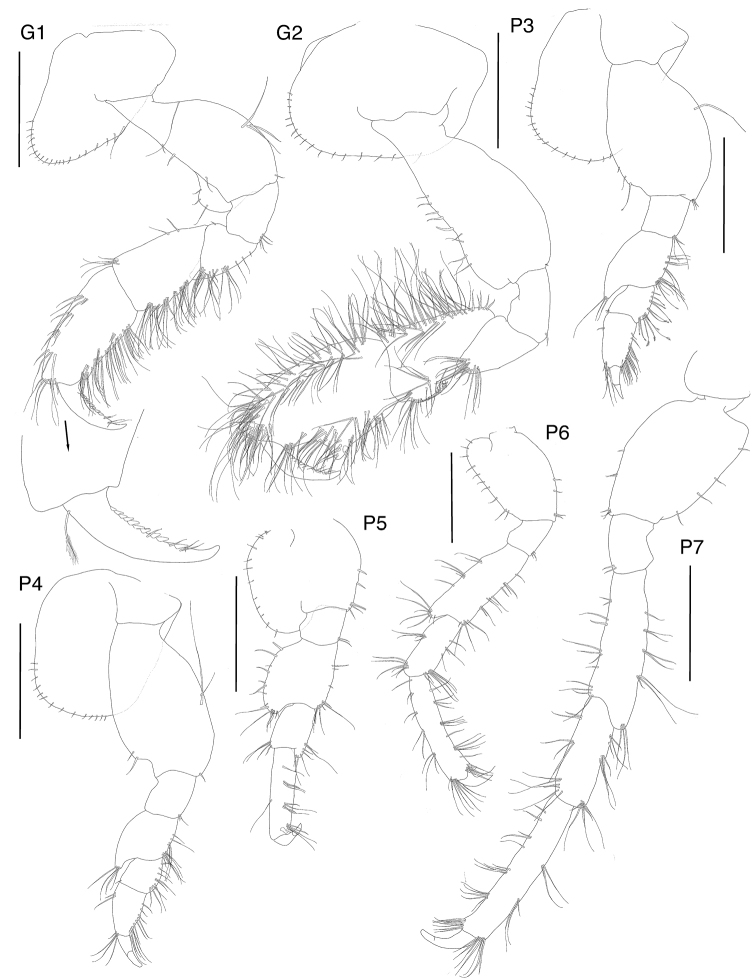
*Pseudopleonexes evensis* sp. n., holotype, 9 mm, male, NIWA 121291, Eve Bay, Wellington, New Zealand. Scale bars: 0.5 mm.

**Figure 8.**
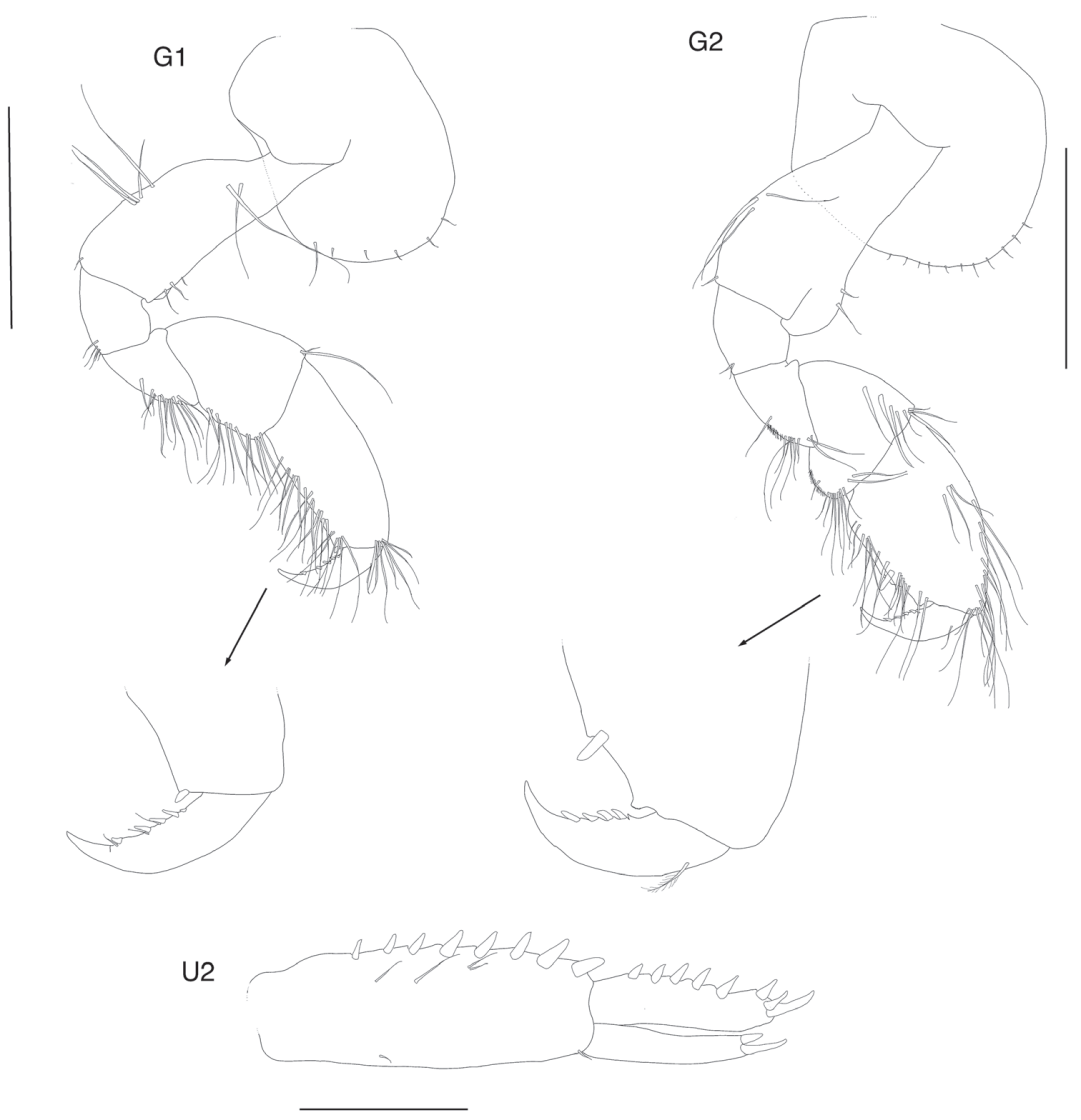
*Pseudopleonexes evensis* sp. n., paratype, female, 7 mm, NIWA 121292, Eve Bay, Wellington, New Zealand. Scale bars: 0.5 mm (**G1–2**), 0.2 mm (**U2**).

######## Remarks.

This relatively rare species can be aligned to J.L. Barnard’s (1972) material and can be identified by the shape of the gnathopods 1 and 2 propodi and palms. The other interesting feature which when first observed in Barnard’s description is the reduced mandibular palp. When the Eve Bay material was collected and dissected it was found to have a very similar mandibular form. If this is a valid character, along with the distinct presence of a setose anterodistal lobe on each of the gnathopod 1 and 2 propodi and the reduced, angled palm of gnathopod 1, validates this material as a new species.

Barnard (1972) described two males one 6.2 mm (E975), one 4.8 mm (E979), and mentioned in the description he only had two specimens and thought they maybe different species based on the presence/absence of the lobe on the uropod 2 peduncle (present on 6.2 mm male/absent on 4.8 mm male), the broadened articles of pereopod 5 (slightly broader than *P. lessoniae*) and the excavation of gnathopod 2 palm (more strongly excavated than *P. lessoniae*). He then mentions three stations where it was collected. The only material that has been able to be found is the 4.8 mm male and this material (when examined) matches to *P. evensis* sp. n. and differs from *P. lessoniae* by the shape and structure of gnathopod 1 (consistent across sizes – 9 mm length described here and the 4.8 mm male he described). The description notes that the uropod 2 peduncular process is absent, however when examined this character is obvioiusly there. The 6.2 mm material was not able to be located and so cannot be verified.

######## Etymology.

The specific name is taken from the name of the type locality, Eve Bay.

######## Distribution.

North Island, New Zealand.

####### Pseudopleonexes lessoniae

Taxon classificationAnimaliaAmphipodaAmpithoidae

(Hurley, 1954)

**Figure 9.**
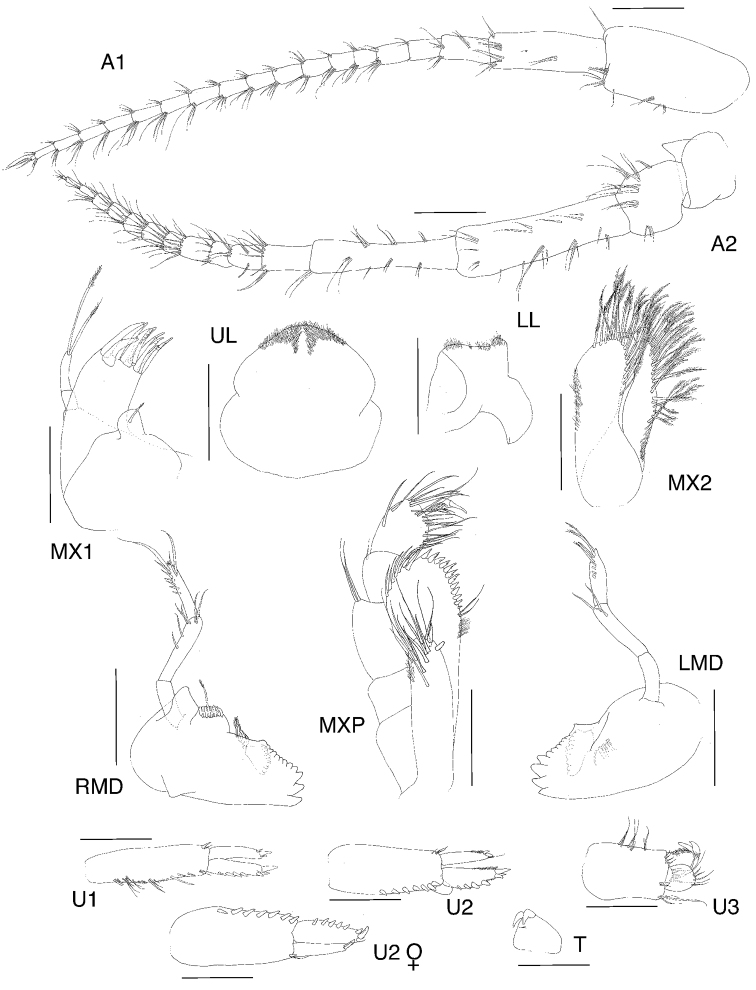
*Pseudopleonexes lessoniae* (Hurley, 1954), male, 9 mm, NIWA 96679, female, 8 mm, NIWA 96679, Breaker Bay Wellington. Scale bars: 0.5 mm.

**Figure 10.**
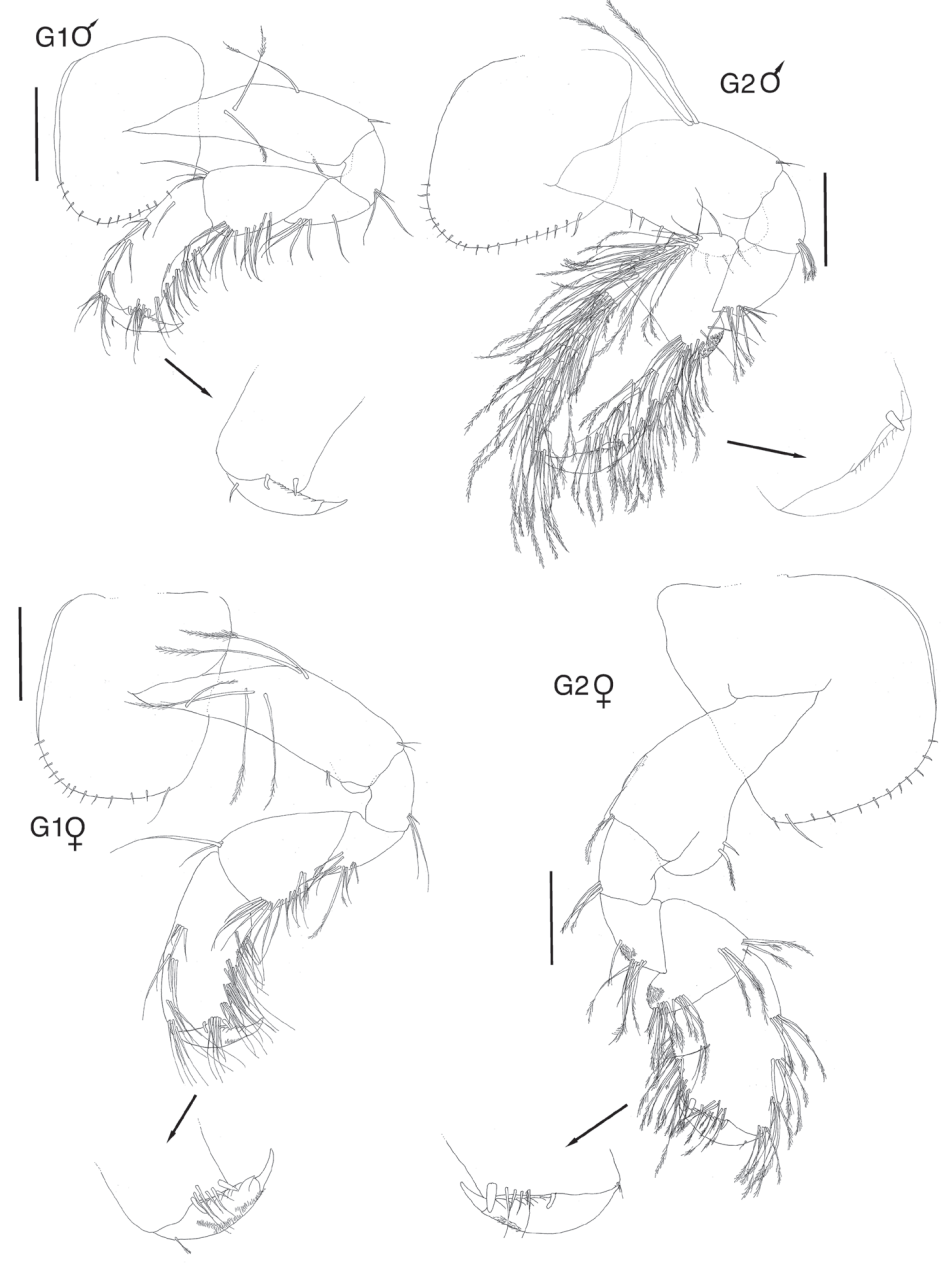
*Pseudopleonexes lessoniae* (Hurley, 1954), male, 9 mm, NIWA 96679, female, 8 mm, NIWA 96679, Breaker Bay Wellington. Scale bars: 0.5 mm.

**Figure 11.**
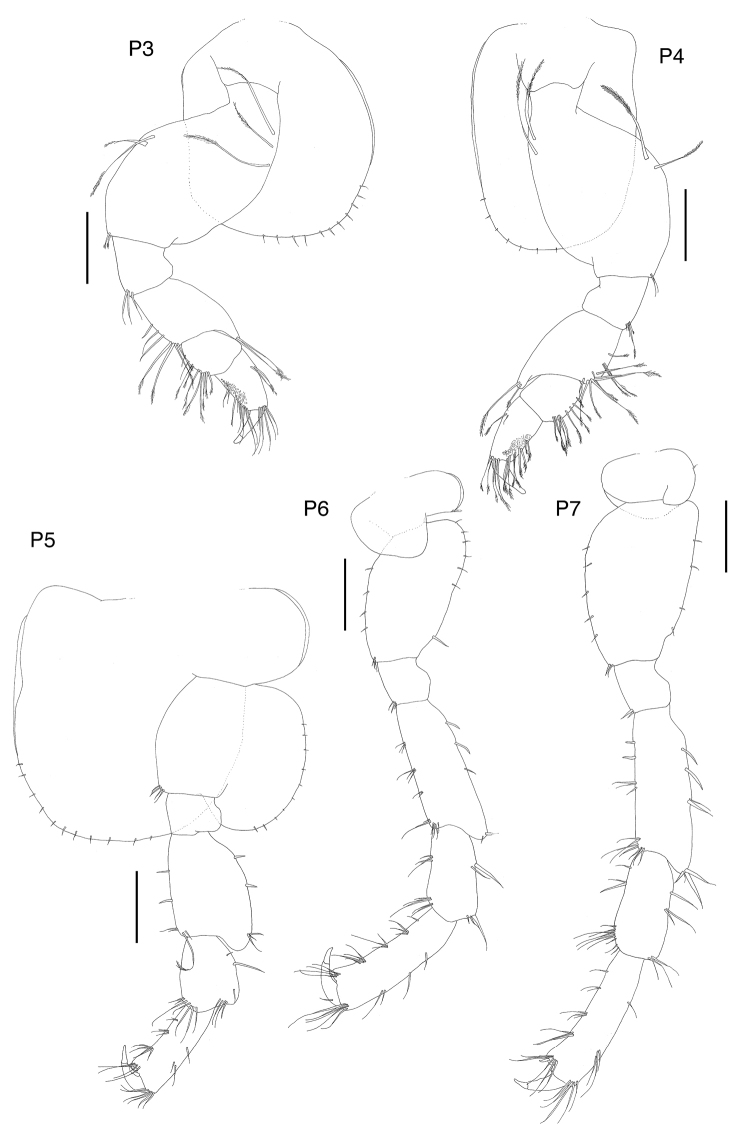
*Pseudopleonexes lessoniae* (Hurley, 1954), male, 9 mm, NIWA 96679, female, 8 mm, NIWA 96679, Breaker Bay Wellington. Scale bars: 0.5 mm.

**Figure 12.**
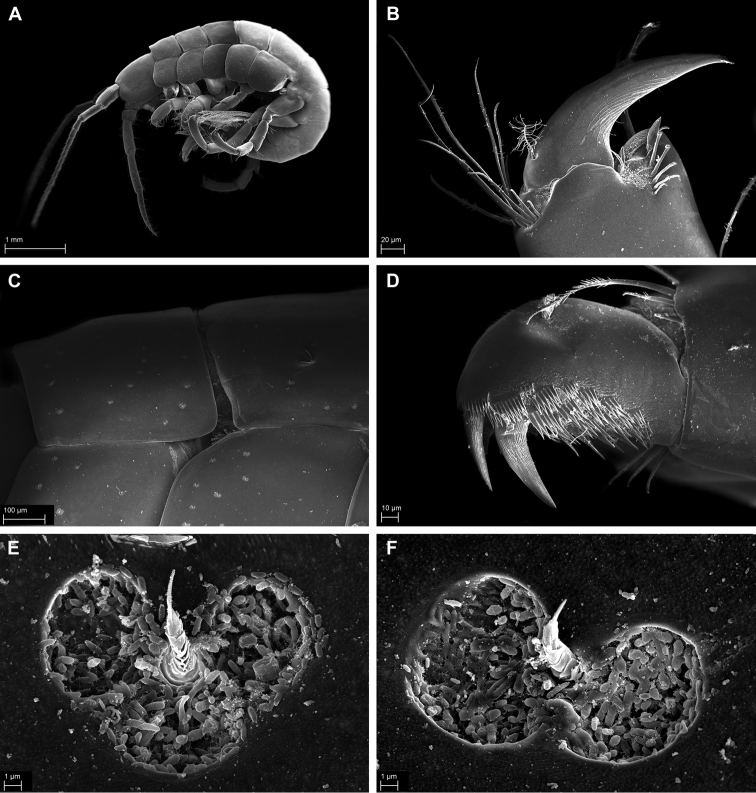
*Pseudopleonexes lessoniae* (Hurley, 1954), male, NIWA 120146, Breaker Bay, Wellington, New Zealand. **A** whole animal, habitus **B** close up of propodus and dactylus pereopod 6 **C** close up of pereon and coxa showing position pits on the surface **D** close up of Uropod 3 rami **E, F** magnification of the heart shaped (**E**) and butterfly shaped (**F**) pits on the coxae and pereon.

Figs 9 , 10 , 11 , 12

Pleonexes lessoniae Hurley, 1954: 620–626, figs. 1–2.
Pseudopleonexes lessoniae .—Conlan 1982: 2020.—Just 2002: 31–40.—Peart 2006: 1–22.
not Ampithoe (Pleonexes) lessoniae.—Barnard 1972: 44, figs 13–14.

######## Material examined.

Holotype: male, 9 mm, NIWA 121308, slide 90 Hurley collection, Island Bay, Wellington, New Zealand, 41°20'39.8"S, 174°46'25.9"E, on *Lessonia variegata*, coll. J.G. Gibbs, 1 August 1950.

Paratype: female, 5.75 mm, NIWA 12309, slide 91, Hurley collection, Island Bay, Wellington, New Zealand, 41°20'39.8"S, 174°46'25.9"E, on *Lessonia variegata*, coll. J.G. Gibbs, 1 August 1950.

All material (other than type material) collected by hand by M. Thiel & A.N. Lörz from *Macrocystis* sp. kelp, Breaker Bay, Wellington, 41.33°S, 174.83°E, 0–1 m between 30^th^ Jan–1^st^ Feb 2013: NIWA 96675–96677, 17 specimens; NIWA 96679, 5 specimens, NIWA 96683–96686, 9 specimens; NIWA 96688–96698, 27 specimens; NIWA 96700–96701, 3 specimens; NIWA 96792–96795, 10 specimens; NIWA 96797–96804, 42 specimens; NIWA 96810 –96818, 59 specimens; NIWA 96819–96820, 15 specimens; NIWA 96822, 14 specimens; NIWA 96824, 7 specimens; NIWA 96826, 1 specimen. NIWA 120146 on SEM stud

######## Diagnosis.

Male: Eye absent (holotype), eyes prominent (additional material examined). Epistome and upper lip, in situ, directed posteriorly around 45°. Antenna 1 longer than antenna 2. Lower lip outer plate slightly notched, almost entire, margin sinusoidal to sometimes flat with larger, subacute corners. Mandible molar reduced and triturating, palp three-articulate, article three distally beaked. Maxilla 1 palp poorly developed and slender tipped with slender plumose setae. Gnathopods strongly setose. Pereopods weakly setose. Gnathopod 1 not sexually dimorphic; coxa not anteroventrally produced; basis anterodistal lobe reduced and rounded bearing on slender seta; propodus subrectangular, anterodistal setal lobe absent, palm transverse, entire, without midmedial tooth, with posterodistal tooth defining palm and one small defining robust seta; dactylus overreaching palm. Gnathopod 2 more robust and larger than gnathopod 1, sexually dimorphic, with long plumose setae on margins; basis anterodistal lobe large and rounded, bearing around 10 robust setae on the margin; carpus subtriangular; propodus longer than carpus; propodus broad, anterodistally setose lobe absent; palm acute, entire (sometimes slightly excavate), midpalmar tooth absent, with small subacute posterodistal tooth defining palm, and one defining robust seta; dactylus subequal to palm. Pereopods 3–4 similar in size and shape, basis expanded and glandular; merus expanded and glandular, forming an acute lobe. Pereopod 5 basis circular, distal articles broad to slender (depending on size), propodus prehensile. Pereopod 6–7 increasing in length, merus and carpus similar widths to propodus (slightly wider), propodi prehensile. Epimeron 3 posteroventral corner rounded without tooth. Uropod 1, in situ, reaching only to the end of uropod 2 peduncle; peduncle distoventral spur absent. Uropod 2 peduncle with large rounded distolateral process. Uropod 3 broad, peduncle without distal robust setae, rami short, outer ramus with two strongly recurved robust setae, patch of denticles; inner ramus with just slender distal setae. Telson subtriangular with strongly recurved cusps, denticles absent, with one slender seta per lobe.

######## Remarks.

Described by Hurley (1954) from Wellington, New Zealand, this species is a small, robust amphipod dwelling in *Lessonia variegata*. Hurley (1954) described it as having similarities to members of the *Ampithoe* group, *Pleonexes*. When the genus *Pseudopleonexes* was constructed (Conlan, 1982), this species was placed as the type of the genus. It has strongly plumose, long setae on the gnathopod 2, a character represented in most of the other species in the genus. Hurley’s types have been located, and are in the NIWA Invertebrate Collection (NIC).

Barnard (1972) assigned two specimens from New Zealand as *P. lessoniae* however, examination of one of the specimens (the other is not locatable) and some confusion in the description indicate that these are not of this species. Comparison with freshly collected material indicates these should be treated as a new species (described above as *P. evensis* sp. n.). *Pseudopleonexes lessoniae* sensu stricto differs from *P. evensis* sp. n. by the absence of an anterodistal setose lobe on the propodi of gnathopods 1 and 2 (strongly present in *P. evensis* sp. n.) and the strongly transverse gnathopod 1 palm, also half the width of the propodus (acute, greatly reduced palm in *P. evensis* sp. n.).

The material described here is from Breaker Bay, very close to the type locality of Island Bay (4.5 km ENE) and was very abundant in *Macrocystis* sp. and until molecular examination is carried out is placed with *P. lessoniae* (Hurley, 1954). The main differences involve the apparent presence/absence of the eye and presence of butterfly and heart-shaped pits and fine hairs covering the pereon and pereopods of the recently collected material (fig. 12).

######## Distribution.

Wellington area, New Zealand.

###### *Sunamphitoe* Spence Bate, 1857

####### Sunamphitoe aorangi

Taxon classificationAnimaliaAmphipodaAmpithoidae

(J.L. Barnard, 1972)

Ampithoe aorangi J.L. Barnard, 1972: 27, 37, figs 8, 9 (part, not 10a–e)
Peramphithoe aorangi .—Shin et al. 2015: 261–264.
Sunamphitoe aorangi .—Peart, 2017: 308
Not Peramphithoe aorangi.—Hughes and Peart 2014: 93–95, fig. 61.

######## Material examined.

Holotype, male, 5.3 mm, NIWA 798, intertidal wash of algae and their rhizomes, Eve Bay, off Strathmore Park, Wellington, New Zealand, 41°19.8’S 174°49.8’E, NZOI Sta E966, coll. J.L. Barnard, 5 Nov 1968.

######## Diagnosis.

Male: Eye prominent. Antenna 1 longer than antenna 2. Epistome and upper lip, in situ, directed straight down, perpendicular to the head. Lower lip outer plate notched, lobes of equal size. Mandible molar triturating, palp with 3 articles, article three distal margin rounded. Maxilla 1 palp well developed. Gnathopods and pereopods weakly setose. Gnathopod 1 coxa slightly produced anteroventrally to form a slight rounded tooth; basis anteroventral lobe reduced and rounded bearing one slender seta; propodus subrectangular, anterodistal setose lobe absent; palm transverse defined by a small robust seta, posterodistal tooth absent; dactylus overreaching the palm. Gnathopod 2 more robust and slightly larger than gnathopod 1, sexually dimorphic, basis anteroventral lobe reduced and rounded with very small setae; carpus subtriangular; propodus longer than carpus; propodus broad, ovoid, anterodistal lobe absent, palm slightly acute (close to transverse), entire, defining posterodistal tooth absent, defining robust seta present; dactylus overreaching the palm. Pereopods 3–4 similar in size and shape; basis expanded and glandular; merus expanded and glandular, lobe subacute. Pereopod 5 basis ovoid to circular, distal articles slightly broadened, propodus weakly prehensile. Pereopods 6–7 similar size, distal articles slender, propodus weakly prehensile. Epimeron 3 posteroventral corner broadly rounded, tooth absent. Uropod 1, in situ, reaching to the end of uropod 2 rami, peduncle acute distoventral spur present. Uropod 2 peduncle rounded lateral distoventral process absent. Uropod 3 narrow, peduncle with distal robust setae absent; outer ramus two strongly recurved robust setae, with patch of lateral denticles; inner ramus with one distal robust seta and slender setae. Telson subtriangular, apical cusps reduced and rounded, no denticles, with both lateral and apical slender setae.

Female. Not documented.

######## Remarks.

This species has recently been redescribed (Peart 2017) and the original material confusion resolved. The differences between this species and the recently described only other species from New Zealand (*S. mixtura* Peart, 2017) are detailed in Table 1 of that publication.

######## Distribution.

Wellington, New Zealand.

####### Sunamphitoe mixtura

Taxon classificationAnimaliaAmphipodaAmpithoidae

Peart, 2017

Sunamphitoe mixtura Peart, 2017: 326
Ampithoe aorangi .—Barnard 1972: 37 (part, sta. E978), fig. 10a–e.

######## Type material.

Holotype: male, 7.8 mm, NIWA 892, small high rock pools, in surf splash zone, lined with filamentous brown alga, Huaroa Point, Whangaparaoa Peninsula (Auckland Province), NZOI Sta. stn E978, coll. J.L. Barnard, 16 Feb 1968.

######## Diagnosis.

Male. Epistome and upper lip, in situ, directed straight down, perpendicular to the head. Lower lip outer plate notched, lobes of equal height. Mandibular molar well developed, triturating; palp with 3 articles, article three rounded distally. Maxilla 1 palp well developed. Gnathopods and pereopods weakly setose. Gnathopod 1 coxa not produced anteroventrally, basis anteroventral lobe medium in size and rounded, bearing three small setae; propodus subrectangular, not produced to form an anterodistal lobe; palm transverse, not defined by a posterodistal tooth, but with a small posterodistal robust seta; dactylus overreaching palm. Gnathopod 2 sexually dimorphic, larger than and more expanded than gnathopod 1; basis anterodistal lobe reduced and rounded bearing three slender setae; carpus very short and subtriangular; propodus much longer than carpus; propodus subtriangular (expanded proximally, narrow distally), anterodistal lobe absent; palm acute, excavate, sculptured, defining posterodistal tooth and robust seta present; dactylus subequal in length to palm. Pereopods 3 basis expanded and glandular; merus expanded with subacute lobe. Perepods 4–7 missing. Epimeron 3 not documented. Uropod 1, in situ, reaching to the end of uropod 2; peduncle with large acute distoventral spur. Uropod 2 peduncle rounded lateral distoventral process absent. Uropod 3 narrow, rami small, peduncle with one distal robust seta; outer ramus with two large recurved robust setae, with patch of lateral denticles; inner ramus with three robust setae and many slender setae. Telson subtriangular, apical cusps small, reduced and rounded, with apical and lateral setae and lateral denticles.

###### Key to the New Zealand species of the family Ampithoidae

**Table:** 

1	Uropod 1 peduncle acute distoventral spur present	**2**
–	Uropod 1 peduncle distoventral spur absent	**3**
2	Gnathopod 2 propodus subtriangular, greatly expanded proximally, narrow distally, palm excavate	***Sunamphitoe mixtura* Peart, 2017**
–	Gnathopod 2 propodus subovoid, broad evenly along length, palm entire	***Sunamphitoe aorangi* (J.L. Barnard, 1972)**
3	Gnathopod 1 palm greatly acute	**4**
–	Gnathopod 1 palm slightly acute or transverse	**6**
4	Gnathopod 2 propodus subrectangular and narrow	**5**
–	Gnathopod 2 subovoid and broad	***Ampithoe hinatore* J.L. Barnard, 1972**
5	Gnathopod 1 propodus broad proximally and narrow distally, palm excavate, prepalmar tooth present, large defining robust seta present	***Exampithoe plumosa* sp. n.**
–	Gnathopod 1 propodus elongated and narrow, palm entire, no defining robust seta	***Exampithoe taylori* Hughes & Peart, 2015**
6	Gnathopods 1 and 2 propodi with setose anterodistal lobes	***Pseudopleonexes evensis* sp. n.**
–	Gnathopods 1 and 2 propodi without setose anterodistal lobes	***Pseudopleonexes lessoniae* (Hurley, 1954)**

## Supplementary Material

XML Treatment for Ampithoe hinatore

XML Treatment for Exampithoe plumosa

XML Treatment for Exampithoe taylori

XML Treatment for Pseudopleonexes evensis

XML Treatment for Pseudopleonexes lessoniae

XML Treatment for Sunamphitoe aorangi

XML Treatment for Sunamphitoe mixtura
